# The impact of smartphone addiction and posture on the prevalence of hand pain among university students

**DOI:** 10.1186/s12889-025-23824-8

**Published:** 2025-10-22

**Authors:** Muhammet Özalp, Mustafa Güven

**Affiliations:** Department of Therapy and Rehabilitation, Kozakli Vocational School, Nevsehir Haci Bektas Veli University, Nevsehir, Turkey

**Keywords:** Smartphone addiction, Hand pain, Students, Posture

## Abstract

**Background:**

Excessive smartphone use among students may cause pain in the hands and fingers. This study aimed to investigate the association between smartphone addiction scores, commonly used smartphone holding postures, and the occurrence of wrist and finger pain in university students.

**Methods:**

This cross-sectional study included 227 university students from Kozaklı Vocational School at Nevşehir Hacı Bektaş Veli University, Turkey. Participants were recruited via voluntary in-class announcements. Data were collected through a demographic questionnaire. The Smartphone Addiction Scale-Short Version (SAS-SV) was used to assess smartphone addiction levels. Participants reported the five most common smartphone holding postures they used (e.g., one-handed texting, two-thumb typing). Pain prevalence, frequency, severity, and impact on daily activities were evaluated using the Cornell Hand Discomfort Questionnaire (CHDQ).

**Results:**

The study revealed that the average age of participants was 19.8 ± 3.07 years. A total of 76.65% reported experiencing pain in one or both hands, with the right hand being the most commonly affected. Higher smartphone addiction scores were significantly associated with increased pain in all evaluated hand areas for both hands (*p* ≤ 0.001). While no statistically significant relationship was found between hand pain and smartphone-holding postures overall, Posture 5 (two-handed use with thumbs) was frequently associated with higher discomfort reports in multiple hand regions, particularly in pinkle, ring, and thumb fingers.

**Conclusion:**

Higher smartphone addiction levels are linked to increased hand pain in students. These findings highlight the need for ergonomic education and preventive interventions to reduce musculoskeletal risks associated with excessive smartphone use.

## Introduction

Smartphone addiction rates among young adults and university students range from about 28% to nearly 80%, depending on the population and criteria used [1–3]. Due to its widespread adoption, there are growing concerns about its impact on physical and mental health.

A significant proportion of smartphone users report musculoskeletal pain localized to the hands, wrists, and digits—particularly the thumb and 2nd–5th fingers—with the highest prevalence observed in the wrist [4–6]. This association is strongly linked to smartphone addiction, as addicted users report significantly higher pain rates than their non-addicted counterparts [3, 7–9]. Mechanistically, prolonged use, especially with poor ergonomic postures (e.g., one-handed gripping with pinky support), exacerbates musculoskeletal strain, increasing pain frequency and severity [4, 10, 11]. Repetitive behaviors such as frequent texting further contribute to chronic thumb pain and reduced pinch-grip strength due to sustained tendon stress [8, 12, 13]. Chronic overuse may also lead to clinical conditions like De Quervain tenosynovitis, as well as functional impairments including diminished dexterity and, in severe cases, structural abnormalities such as finger deformities [10, 14, 15]. Demographic analyses reveal that women, younger individuals, and students—groups with high smartphone engagement—are disproportionately affected [7, 9, 16].

The effects of smartphone posture on hand function are significant, impacting pain, grip strength, and overall hand functionality. Various studies have explored how different smartphone usage postures contribute to these effects. Smartphone use, particularly with poor wrist and elbow angles, is associated with increased pain in the hand and upper body. This is exacerbated by smartphone addiction, which correlates with higher pain levels and reduced hand function [5, 17, 18]. Different smartphone postures, such as lying down or using one hand with pinky support, can lead to musculoskeletal pain, particularly in the wrist, thumb, and fingers. The prone position and flexed neck posture are notably associated with increased pain [5, 18]. Prolonged one-handed smartphone use (especially with thumb interaction) increases pain in the thumb and thenar muscles [19]. Thumb-dominated postures during texting cause higher muscle activation in the flexor pollicis longus and abductor pollicis brevis, leading to fatigue and reduced thumb mobility [20]. Students who used smartphones > 5 h/day had significantly weaker grip strength and higher wrist pain prevalence, especially with one-handed postures [21]. Two-handed postures reduce thumb strain but increase wrist deviation, while one-handed use leads to higher thenar muscle fatigue [22].

Despite growing evidence linking smartphone use with hand pain, there remains a clear gap in the literature regarding which specific hand regions are most affected and how different smartphone-holding postures contribute to musculoskeletal pain. To address this gap, the present study aims to investigate the relationship between smartphone addiction scores, holding postures, and hand pain among university students.

## Materials and methods

This research is a descriptive and cross-sectional study conducted among students at Kozaklı Vocational School of Nevşehir Hacı Bektaş Veli University during the spring semester of the 2024–2025 academic year. The total student population at the school was 550. The required sample size was calculated as 227 using the formula: n = [Nt2pq]/[d2(N-1) + t2 pq], where N = 550, *p* = 0.5, q = 0.5, d = 0.05 and t = 1.96.

A convenience sampling approach was used, where students who met inclusion criteria and were present during classroom announcements were invited to voluntarily participate in the study. A total of 232 students initially agreed, but 5 were excluded based on the exclusion criteria, resulting in a final sample of 227.

Inclusion criteria included: students aged between 18 and 25 years, enrolled in the school, and willing to participate voluntarily. Exclusion criteria were: a prior history of hand surgery, carpal tunnel syndrome, inflammatory arthritis, or wrist fractures or soft tissue injuries within the preceding six months.

Out of the 232 students who initially expressed willingness to participate, five were excluded based on the exclusion criteria, resulting in a final sample of 227 participants. Specifically, one individual was excluded due to a history of hand surgery, three due to recent trauma within the past six months, and one due to a wrist fracture.

### Ethics

This research followed ethical guidelines outlined in the Declaration of Helsinki. The study protocol received approval from Nevşehir Hacı Bektaş Veli University’s Non-Interventional Clinical Research Ethics Committee (Decision #2025.03.11, dated March 26, 2025). All participants provided written informed consent before taking part in the study.

### Data collection

Data were collected during face-to-face interviews led by the researcher. Before participating, individuals received full explanations about the study’s purpose and procedures, and provided either written or verbal consent. The interviews took place over a two-week period at Kozakli Vocational School. Each questionnaire session lasted approximately 10 min and was conducted individually with researcher supervision to ensure data accuracy and completion.

### Demographic questionnaire and hand posture assessment

A demographic questionnaire was used to record participants' age, dominant hand, academic program, and eligibility criteria. For posture classification, ten smartphone-holding postures were selected based on Banadaki et al. [19]. Each posture was illustrated with a labeled image, and participants were asked to select the posture they most frequently used. However, only five postures were chosen by participants. The remaining five were not selected, likely due to their relative complexity or lower prevalence in everyday usage habits. The five adopted postures were:

- Bimanual asymmetric grip – left hand stabilizes the phone while the right thumb interacts with the screen.
- Unimanual top interaction – right hand holds the phone (four fingers behind), right thumb reaches upper screen.
- Unimanual mid/low interaction – right hand supports the phone (with pinky base support), right thumb reaches center/lower screen.
- Contralateral unimanual interaction – left hand holds phone, left thumb interacts.

**Fig. 1:**
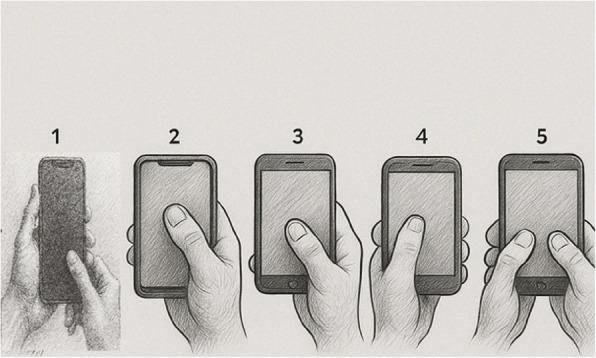
The five most commonly observed smartphone holding postures among participants. Different holding postures 1,2,3,4, and 5 for using the smartphone: **1** Bimanual asymmetric grip - left hand stabilizes the device while right thumb performs screen contact; **2** Unimanual top interaction - right hand grasps the device (all four fingers positioned dorsally) with thumb operating the upper screen region; **3** Unimanual mid/low interaction - right hand supports the device (using fifth digit as base) while thumb accesses central or inferior screen areas; **4** Contralateral unimanual interaction - left hand supports device with ipsilateral thumb engaging mid or lower screen zones; **5** Bimanual symmetric interaction - both hands stabilize the device in portrait orientation with bilateral thumb input

Bimanual symmetric interaction – both hands hold phone in portrait mode, both thumbs used. (Fig. 1 shows the five most commonly observed smartphone holding postures among participants).

Originally developed by Kwon et al., the scale contains 10 items rated on a 6-point likert scale (1 = strongly disagree, 6 = strongly agree). Total scores range from 10 to 60. We used the Turkish-adapted and validated version by Noyan et al. [23]. Participants were classified as “addicted” if their scores met or exceeded gender-specific cut-off points: 33 for females and 31 for males, as used in the original validation.

Cornell Hand Discomfort Questionnaire (CHDQ): The study used a concise 6-item questionnaire divided into three sections. Participants first viewed a hand diagram highlighting six specific areas, then answered questions about: How often they experienced hand pain/discomfort in the past week, the severity of any pain, whether it interfered with their daily activities. Scores were calculated by multiplying frequency, discomfort level, and disability impact (frequency × discomfort × disability). Each hand area could score up to 90 points, with a maximum total score of 560 across all six areas—higher scores indicating more severe discomfort. For prevalence analysis, we considered any reported pain in at least one hand area as"yes"while no pain in any area was"no". This Turkish version of the questionnaire (CHDQ) was validated by Dr. Oğuzhan Erdinç [24]. Although we did not re-assess internal consistency for this study sample, previous validation reported high reliability.

### Statistical analysis

In data analysis, Uni-Variable logistic regression test was used to investigate the relationship between smartphone addiction score and discomfort prevalence in six areas of each hand. Kruskal–Wallis test was used to analyze the relationship between total discomfort scores (calculated by multiplying frequency, intensity and interference scores) for each area and different holding patterns. Additionally, Kruskal–Wallis test was used to investigate the relationship between the total pain count for each hand in different holding patterns.

## Results

A total of 227 university students participated in this study, with the majority being female (87.67%). The mean age was 19.8 years (± 3.07). Among participants, 54.19% were classified as smartphone-addicted based on the SAS-SV. Regarding smartphone usage habits, 50.66% of students reported using both hands while holding or interacting with their devices, whereas 45.81% used only the right hand and 3.52% used only the left. The prevalence of hand pain was reported as follows: 23.35% of students reported no pain, 34.36% reported pain in the right hand, 2.64% in the left hand, and 39.65% in both hands (Table 1).

**Table 1:** Quantitative and qualitative variables of student participants (*n* = 227), dominant hand, pain symptoms prevalence, and smartphone addiction status

Variable		Mean ± SD	Min–Max
Age		19.82 ± 3.07	17–47
Height (cm)		164.49 ± 7.78	150–190
Weight (kg)		59.19 ± 10.78	36–95
The sum of the total discomfort scoring in the right hand (Cornell)		16.5 ± 32.5	0–330
The sum of the total discomfort scoring in the left hand (Cornell)		10.34 ± 45.6	0–560
Number of discomfort symptoms in the right hand (Cornell)		2.14 ± 1.81	0–6
Number of discomfort symptoms in the left hand (Cornell)		1.07 ± 1.63	0–6
**Variable**	**Classification**	**Frequency**	**Percentage**
Gender	Male	28	12.33
	Female	199	87.67
Smartphone addiction	Addicted	123	54.19
	Non addicted	104	45.81
The hand used to work with the smartphone	Right	104	45.81
	Left	8	3.52
	Both	115	50.66
Academic Program	Physiotherapy	169	74.45
	Occupational Therapy	52	22.91
	Social Work	6	2.64
Prevalence of pain	No pain	53	23.35
	Right hand	78	34.36
	Left hand	6	2.64
	Both hands	90	39.65

According to the Cornell Hand Discomfort Questionnaire (CHDQ), the total discomfort scores were higher in the right hand (mean = 16.5 ± 32.5) compared to the left hand (mean = 10.34 ± 45.6). Similarly, the average number of reported pain symptoms was greater in the right hand (2.14 ± 1.81) than the left hand (1.06 ± 1.64). Although a higher frequency of discomfort was observed in the right hand compared to the left, this comparison was descriptive in nature and not based on statistical testing. (Table 1).

**Fig. 2:**
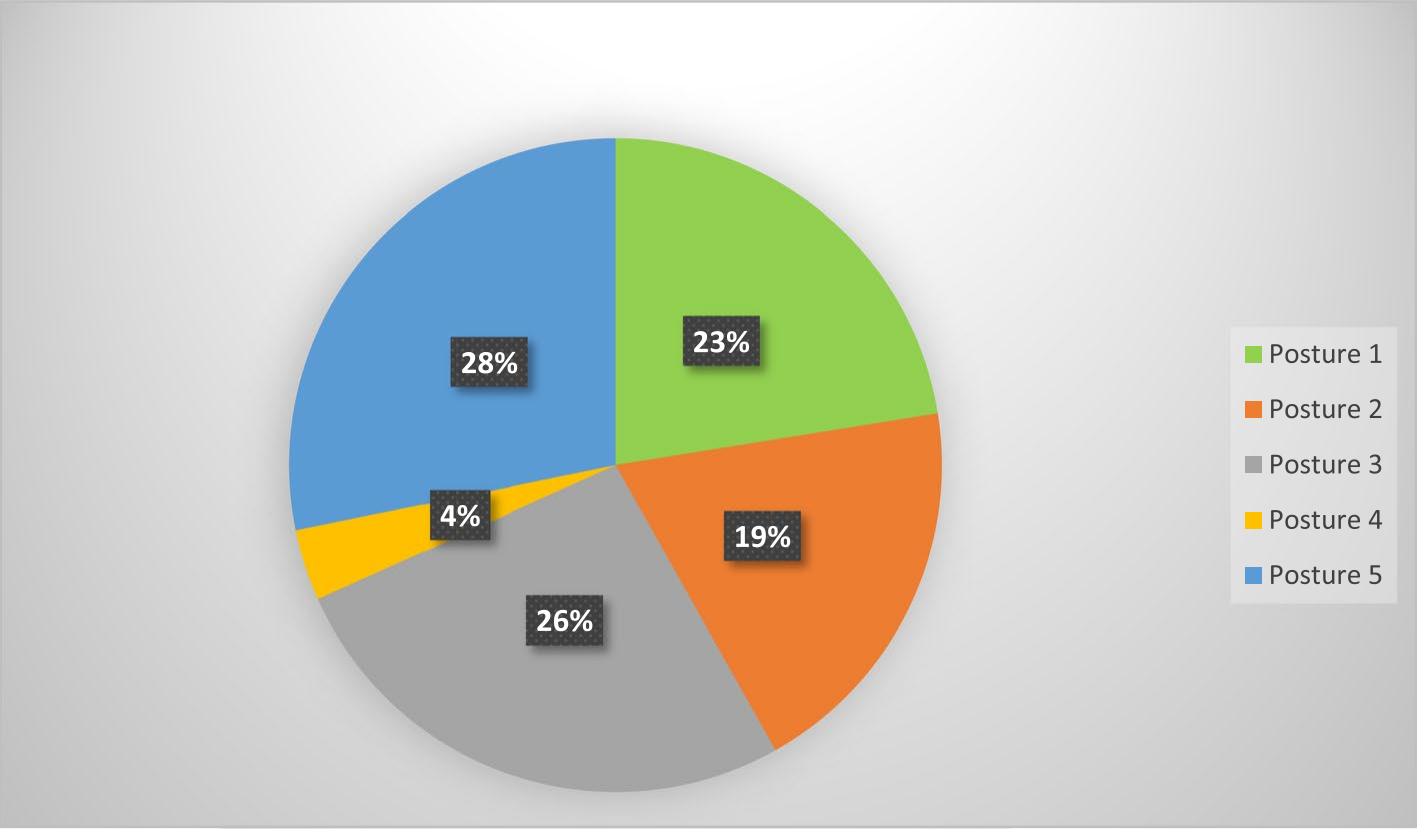
Percentage of posture types used for holding smartphones among participating students (*n* = 227). Posture 1 (%23): left hand stabilizes the device while right thumb performs screen contact; Posture 2 (%19): right hand grasps the device (all four fingers positioned dorsally) with thumb operating the upper screen region; Posture 3 (%26): right hand supports the device (using fifth digit as base) while thumb accesses central or inferior screen areas; Posture 4(%4): left hand supports device with ipsilateral thumb engaging mid or lower screen zones; Posture 5 (%28): both hands stabilize the device in portrait orientation with bilateral thumb input

Figure 2 reveals that the most prevalent holding posture when using a smartphone with the right hand is posture five, which both hands to hold with both thumbs to touch in portrait orientation. This posture was reported by 28% of participants (Fig. 2 shows the distribution of smartphone holding postures among participants).

Univariate logistic regression analysis revealed that higher smartphone addiction scores were significantly associated with an increased risk of pain across all evaluated hand areas for both right and left hands (*p* ≤ 0.001) (Table 2).

**Table 2:** The results of the impact of smartphone addiction score on the prevalence of pain using uni-variable logistic regression

Risk Factors	Hand area	OR	95% CI	p
Smartphone addiction score	Right Area A	1.07	1.04–1.10	< 0.001
	Right Area B	1.06	1.03–1.09	< 0.001
	Right Area C	1.09	1.06- 1.12	< 0.001
	Right Area D	1.08	1.05–1.11	< 0.001
	Right Area E	1.08	1.05–1.11	< 0.001
	Right Area F	1.07	1.04–1.10	< 0.001
	Left Area A	1.07	1.04–1.10	< 0.001
	Left Area B	1.07	1.04–1.10	< 0.001
	Left Area C	1.08	1.05–1.12	< 0.001
	Left Area D	1.07	1.04–1.11	< 0.001
	Left Area E	1.07	1.04–1.11	< 0.001
	Left Area F	1.03	1.00–1.06	0.016

However, the Kruskal–Wallis test showed no statistically significant relationship between smartphone-holding postures and discomfort in most hand regions, except for Area B of the left hand, which showed a borderline significant relationship (*p* = 0.05) (Table 3).

**Table 3:** The relationship between holding a smartphone in the hands and experiencing discomfort in different areas of the hands using the Kruskal Wallis test

Variable Independent	Posture 1,2,3,5 (Right hand or both hands), df = 3		Posture 1,4,5 (Left hand or both hands), df = 2	
Variable Dependent	h	p	h	p
Area A	1.97	0.57	1.17	0.55
Area B	2.04	0.56	6.00	0.05
Area C	2.22	0.52	1.13	0.56
Area D	5.19	0.15	1.55	0.45
Area E	1.82	0.60	0.65	0.72
Area F	1.94	0.58	1.07	0.58

Kruskal–Wallis H test used to compare pain scores across posture groups. Degrees of freedom (df) = 3 for right or both hand group, and df = 2 for lef tor both hand group

Frequency data from Table 4 suggest that Posture 5 is associated with the highest number of reported discomfort instances in multiple areas for both hands, particularly in Areas B and C (Table 4).

**Table 4:** Number of discomfort felt in different areas of the hands while holding a smartphone

		Posture 1	Posture 2	Posture 3	Posture 4	Posture 5
Right hand	Area A	15	16	15	0	21
	Area B	23	20	31	4	31
	Area C	23	19	23	3	32
	Area D	17	8	13	2	22
	Area E	17	19	21	2	18
	Area F	19	10	19	0	22
Left hand	Area A	11	7	7	1	18
	Area B	10	6	5	3	25
	Area C	11	6	3	1	16
	Area D	10	4	3	3	15
	Area E	8	6	3	1	13
	Area F	12	8	6	0	15

## Discussion

This study identified a consistent association between higher smartphone addiction scores and increased hand discomfort among university students. Participants with elevated addiction scores reported significantly more discomfort in all six hand regions assessed. These findings align with previous research indicating that excessive smartphone use is linked to musculoskeletal symptoms, particularly in the thumb, wrist, and fingers [3, 5, 7, 12, 16, 17, 25–27]. Students classified as addicted to smartphones have been shown to be two to three times more likely to report hand discomfort compared to non-addicted peers [7]. The most commonly affected areas are the wrist, thumb, and fingers, with pain and discomfort often linked to repetitive and prolonged smartphone use [3, 25].

In the present study, pain scores increased significantly across all six anatomical regions (A–F) of both hands in parallel with rising addiction scores. This reinforces earlier findings that prolonged smartphone use—especially activities involving intensive thumb motion and sustained wrist positioning—is a contributing factor in hand discomfort [19, 28, 29]. The most affected areas included the thumb (C), thumb base (E), and wrist (F), which are subject to repetitive strain and mechanical overload during common smartphone activities such as texting, swiping, and scrolling. These regions also align with previously reported biomechanical vulnerabilities, where thumb flexor and abductor muscles (e.g., flexor pollicis brevis, abductor pollicis brevis, opponens pollicis) are activated repetitively during prolonged use [20, 30–32]. Dominant hand use appears to play a critical role in discomfort distribution. Consistent with prior findings, our data showed that right-hand discomfort was more prevalent than left, which is likely due to the majority of participants being right-handed. This observation is supported by Abdulahi et al. and Amjad et al., who found that dominant hand involvement in prolonged phone use contributed to increased pain, especially in the wrist [21, 33]. Notably, our study adds to the existing literature by linking not only usage duration but also smartphone addiction scores—which incorporate behavioral components such as compulsive use and emotional dependence—to regional hand discomfort. This extends beyond simple screen time, suggesting that psychological dependence may exacerbate physical strain.

Smartphone-holding posture was another key factor. The analysis revealed a borderline significant association between smartphone holding postures and discomfort in Area B of the left hand (p = 0.05), suggesting a possible link worth further investigation. Although this relationship did not reach conventional levels of statistical significance for the right hand, the findings highlight that specific postures may differentially affect discomfort in certain hand regions. Across all postures, the highest frequency of discomfort was reported in Areas B (4th–5th fingers) and C (thumb), particularly in Postures 3 and 5. Posture 3, characterized by one-handed use with pinky support, was associated with increased reports of discomfort in the ulnar region (Area B) and thumb (Area C) of the right hand. This grip likely places excess mechanical load on the ulnar side of the hand due to device weight being distributed toward the pinky finger a finding previously noted by Wang et al.[34].

Posture 5, involving bilateral thumb interaction, was also frequently linked to discomfort in both the thumb (C) and little finger region (B), particularly on the right side. Although some literature suggests that two-handed use may reduce strain [34], this discrepancy may be explained by differences in posture duration, screen size, or grip force. In our study, Posture 5 was the most commonly adopted (used by 115 participants), and its frequent use may have contributed to the increased pain reports.

Findings from Banadaki et al. are particularly relevant [19]. Their study indicated that 25.4%–29.6% of students experienced discomfort in the right thumb, thumb base, and wrist—regions that were also commonly affected in our study. They also noted that 64% of participants primarily used their right thumb, aligning with our data showing dominant right-hand discomfort patterns. This parallel reinforces the role of repetitive thumb activity and wrist positioning in the development of hand strain [19, 30, 33, 34].

Taken together, our findings underscore the biomechanical consequences of prolonged smartphone use and the cumulative strain imposed by both grip posture and addiction-related usage behavior. The anatomical concentration of discomfort in the thumb, ulnar fingers, and wrist regions reflects the muscular and joint stress associated with frequent smartphone interaction. These results are further supported by prior work from Gustafsson, Sharan, and Baabdulla, who demonstrated that overuse of the dominant hand in smartphone interaction leads to upper limb fatigue and musculoskeletal pain [13, 35–37].

The present findings have practical implications for multiple stakeholders. For students, increased awareness of ergonomic principles and posture-related risks could help mitigate discomfort. Educational campaigns promoting regular breaks and balanced grip use may reduce the incidence of smartphone-related pain. Clinicians should consider evaluating smartphone usage habits and addiction levels when assessing hand or wrist complaints in young adults. Additionally, ergonomic designers may use these insights to develop phone accessories and grip aids that reduce biomechanical stress during prolonged use.

### Limitations

While this study provides valuable insights, several limitations warrant consideration. First, the reliance on self-reported data may introduce biases, as participants could inaccurately recall their smartphone usage or subjectively interpret their pain levels. Second, variations in smartphone characteristics (e.g., size, weight, or design) were not accounted for, though these factors likely influence how devices are held and the resulting strain on the hand. Additionally, the study was conducted at a single vocational school, which may limit the generalizability of the findings to broader university student populations. Further, individual physical differences—such as hand size and grip strength, which likely affect posture and susceptibility to discomfort were not measured. Moreover, the gender composition of the sample was skewed toward females, potentially restricting the applicability of the results to more gender-balanced academic settings.

Another limitation is the absence of certain potential confounding variables, such as the duration of smartphone use, which were not collected during data gathering. Furthermore, multivariable analysis methods (e.g., logistic regression) were not employed to control for other confounders like age and gender. Although the statistical techniques used were appropriate for the study’s aims, the lack of adjustment for these factors may limit the interpretation of the independent effects of specific variables on hand discomfort. Future studies are encouraged to include a broader range of variables and apply multivariable models to enhance analytical robustness.

## Conclusion

This study demonstrates a significant association between smartphone addiction and musculoskeletal discomfort in the hand, particularly in the thumb, wrist, and ulnar finger regions. The findings indicate that both behavioral factors (e.g., addiction severity) and mechanical factors (e.g., holding posture) contribute to the development of localized pain and discomfort among university students.

Given the repetitive and forceful movements involved in smartphone use—especially those performed by the dominant thumb—prolonged and uninterrupted device handling may lead to cumulative strain injuries. Preventive strategies such as limiting continuous usage time, incorporating regular breaks (e.g., every 20 min), and utilizing supportive accessories (e.g., smartphone stands) may help reduce biomechanical stress on the hand.

Users are also encouraged to alternate finger use during typing, reduce dependence on one-handed grips, and consider voice-to-text functions to minimize thumb overload. These practical adjustments may contribute to healthier smartphone interaction and reduce the risk of long-term musculoskeletal complications.

From a public health and educational standpoint, the results underscore the importance of raising awareness among young adults about the ergonomic risks of excessive smartphone use. Universities and healthcare professionals should consider integrating digital ergonomics education into student wellness initiatives to prevent smartphone-related injuries and promote sustainable technology habits.

## Data Availability

The data that support the findings of this study are not openly available due to reasons of sensitivity and are available from the corresponding author upon reasonable request.

## Abbreviations

SAS-SV: Smartphone Addiction Scale-Short Version
CHDQ: Cornell Hand Discomfort Questionnaire
